# Retinal oxygen saturation, vessel diameter and flicker response in eyes with specific subtypes of neovascular age-related macular degeneration during aflibercept treatment

**DOI:** 10.1371/journal.pone.0271166

**Published:** 2022-07-12

**Authors:** Stefan Sacu, Katharina Eibenberger, Doreen Schmidl, Sandra Rezar-Dreindl, Gerhard Garhöfer, Jonas Brugger, Wolf Buehl, Leopold Schmetterer, Ursula Schmidt-Erfurth

**Affiliations:** 1 Department of Ophthalmology, Medical University of Vienna, Vienna, Austria; 2 Department of Clinical Pharmacology, Medical University of Vienna, Vienna, Austria; 3 Department of Medical Statistics, Medical University of Vienna, Vienna, Austria; 4 Center for Medical Physics and Biomedical Engineering, Medical University of Vienna, Vienna, Austria; 5 Singapore Eye Research Institute, Singapore, Singapore; Public Library of Science, UNITED STATES

## Abstract

**Purpose:**

To evaluate the effect of intravitreal aflibercept monotherapy on arterial and venous oxygen saturation, retinal vessel diameter and flicker response in patients with newly diagnosed specific subtypes of exudative maculopathy.

**Methods:**

This prospective study included forty-four eyes of 44 patients with treatment-naïve polypoidal choroidal vasculopathy (PCV, n = 12), hemorrhagic choroidal neovascularization (hCNV, n = 12), pigment epithelium detachment (PED, n = 9) and type 3 MNV (RAP, n = 11). All patients received three initial aflibercept 2mg/0.05ml injections (Eylea^®^) in monthly intervals (loading phase) and were subsequently treated until month 12. Measurements of arterial and venous oxygen saturation, vessel diameters and flicker response were performed using the Dynamic Vessel Analyzer (DVA; IMEDOS, Jena, Germany). Statistical analysis was performed on the total population at baseline, after loading dose and at the last follow-up visit.

**Results:**

The arterial oxygen saturation was 94.01±2.14% and showed no change after loading dose (93.94±2.88%, p = 0.4; estimated difference [confidence interval] -0.38 [-1.24; 0.48]) and at the last visit (95.48±1.90%; p = 0.1; -1.29 [-0.34; 2.91]). The venous oxygenation during treatment was 78.49±6.93% at baseline, 80.94±7.71% after 3-monthly injections (p = 0.7; -0.43 [-2.72; 1.86]) and 80.56±7.33% at month 12 (p = 0.5; 1.07 [-2.10; 4.24). The arterial and venous vessel diameters were 94±22μm and 131±19μm at baseline, and remained unchanged following aflibercept loading dose and at the last follow-up visit (p-value: p = 0.5; 2.30 [-5.00; 9.59] p = 0.8; 0.59 [-3.17; 4.34]). During stimulation with flicker light, arterial diameter changed by +1.24±4.93% at baseline and remained stable at month 3 (+2.70±5.95%; p = 0.5; 1.43 [-2.54; 5.41]) while the change in venous diameter during flicker stimulation was +4.52±4.45% at baseline and +4.13±3.65% after loading dose (p = 0.4, 5.18 [1.73; 8.63]).

**Conclusion:**

During intravitreal aflibercept treatment oxygen saturation, vessel diameter and flicker response did not change in the total population of patients with specific subtypes of exudative maculopathy.

## Introduction

The multifactorial pathogenesis of neovascular age-related macular degeneration (nAMD) involves chronic oxidative stress, inflammatory pathways, ischemia and hypoxia. An abnormal retinal oxygen metabolism exists in patients with neovascular AMD in comparison to the healthy population, with a reduction of oxygen extraction and reduced oxygen supply in the tissue [1]. Several studies have provided evidence suggesting a decrease of choroidal and retinal blood flow in AMD [2–4]. The presence of an abnormal choroidal circulation and the corresponding ischemia and/or hypoxia results in the expression of various mediators and the development of angiogenesis, ultimately impairing the visual function of the patients. In a study evaluating patients with type 3 lesions, expression of HIF-1 alpha and HIF-2 alpha was shown in vascular endothelial cells of the neovascularization suggesting a hypoxic status in patients with retinal type 3 MNV [5]. However, limited evidence exists about the oxygen metabolism in those patients with the various specific subtypes of neovascular AMD.

The classification of neovascular lesions which are localized below the retinal pigment epithelium (RPE) include type 1 macular neovascularization (MNV), corresponding to occult lesions and subretinal lesions or type 2 MNV, corresponding to classic choroidal neovascular (CNV) lesions. Besides these lesions, development of new vessels within the retina is classified as a type 3 MNV, known as RAP [6]. In addition, polypoidal choroidal vasculopathy (PCV), a disease of the choroidal vasculature, is thought to be related to type 1 MNV. Subtypes of neovascular lesions caused by age-related macular degeneration (AMD) can be further classified into predominantly hemorrhagic choroidal neovascular lesions and lesions with predominant detachment of the retinal pigment epithelium. These lesions, including type 3 MNV, polypoidal choroidal vasculopathy (PCV), hemorrhagic CNV (hCNV) and pigment epithelium detachment (PED) are recognized to be variants or subforms of nAMD and are all associated with a poor functional prognosis and treatment response [7–11].

Evaluation of the oxidative parameters was also done during the treatment of patients with anti-vascular endothelial growth factor (anti-VEGF) agents, which has shown to be effective in the treatment of nAMD. Significant vasoconstriction of the retinal arterioles was demonstrated in studies during anti-VEGF monotherapy. This effect was explained by the inhibition of VEGF, which functions as a potent vasodilator via nitric oxide activation. These studies were mainly evaluating the impact of ranibizumab or bevacizumab in patients with neovascular AMD [12–14]. However, evidence about the oxygen saturation in patients with subtypes of nAMD during treatment with aflibercept is lacking. Because of the widespread use of anti-VEGF agents, their effects on ocular oxygenation needs to be further explored, in order to gain more insight into the retinal hemodynamic consequences of anti-VEGF treatment. Therefore, the aim of this study was to evaluate possible changes of retinal oxygen saturation in patients with different subtypes of nAMD following intravitreal aflibercept loading dose. In addition, retinal vessel diameters and neurovascular coupling as the response of retinal vessel diameters to stimulation with flicker light were assessed. Furthermore, measurements of oxygen saturation, flicker response and vessel diameter were repeated after one year of intravitreal aflibercept monotherapy.

## Methods

The study adhered to the Declaration of Helsinki and all patients gave written informed consent before inclusion. Approval was gained from the local ethics committee of the Medical University of Vienna and the Austrian Federal Office for Safety and Health Care and registration was performed at clinicaltrialsregister.eu (Eudra CT Nr: 2014-002384-15).

**Fig 1:**
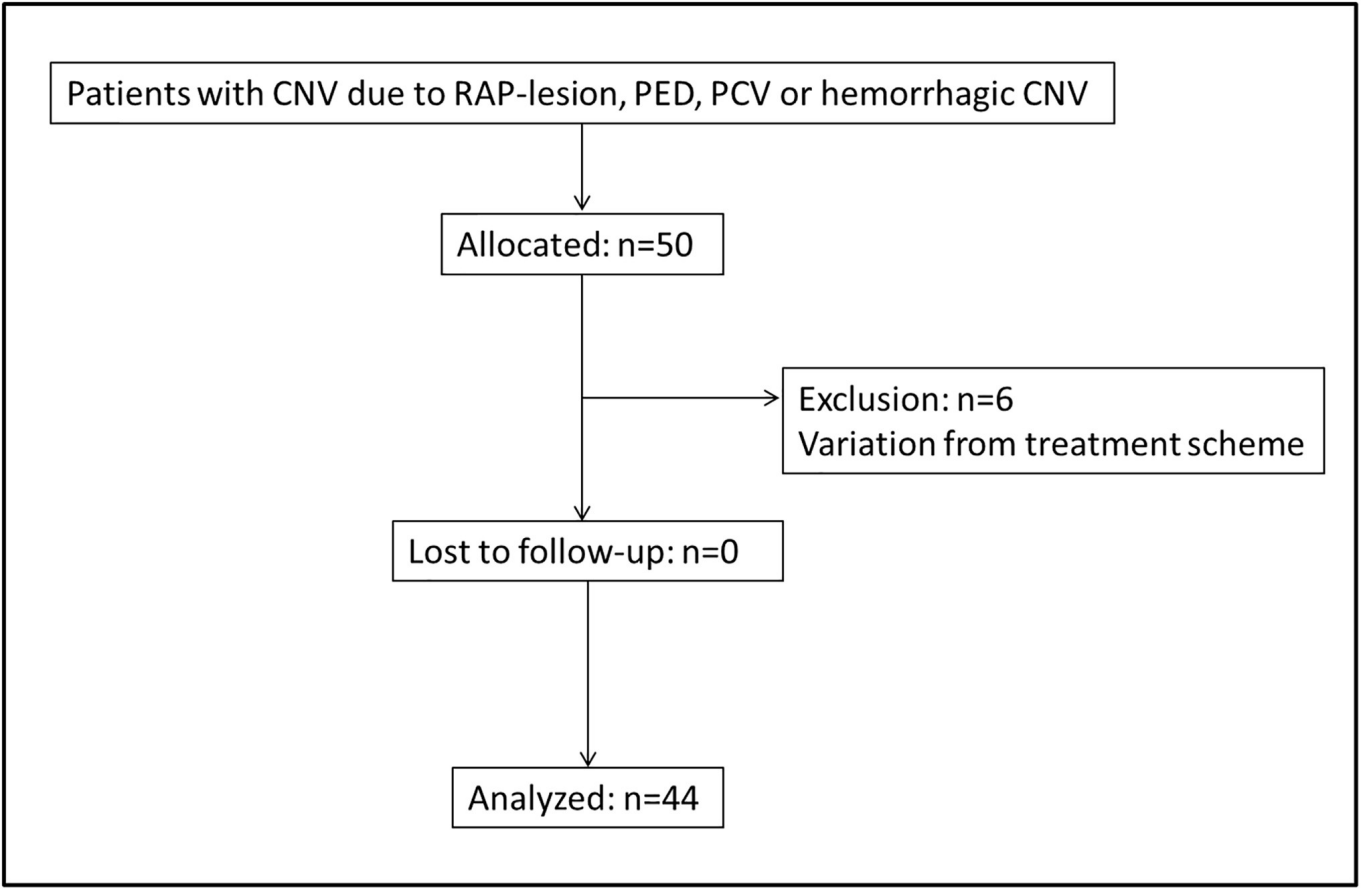
Flow chart.

In this prospective, non-randomized clinical trial a total of 50 patients with CNV due to type 3 MNV lesion, PED, PCV or hemorrhagic CNV (hCNV) were included. Patients were selected at the Department of Ophthalmology, Medical University of Vienna, Austria between October 2014 and November 2016. Inclusion criteria comprised newly diagnosed treatment naïve eyes with evidence of one of the following subtypes of exudative maculopathy (PCV, hCNV, PED or type 3 MNV), best-corrected visual acuity (BCVA) better than 20/400 and an age above 50 years. All patients meeting the inclusion criteria received an initial loading dose of three injections in monthly intervals of aflibercept (Eylea^®^ 40mg/ml; Bayer AG, Leverkusen, Germany), under sterile conditions and following national guidelines. Treatment was then administered in bimonthly intervals, whereas follow-up visits were scheduled monthly up to month 12. In 6 patients the treatment scheme differed from the protocol with the need of more re-treatments due to increased disease activity. Hence, these patients had to be excluded from the analysis. Therefore, 44 patients with polypoidal choroidal vasculopathy (PCV, n = 12), hemorrhagic choroidal neovascularization (hCNV, n = 12), pigment epithelium detachment (PED, n = 9) and retinal angiomatous proliferation (type 3 MNV, n = 11) were included for analysis. The flow chart of the study is shown in Fig 1.

Exclusion criteria comprised of any previous treatment for exudative maculopathy, including photodynamic therapy, intravitreal anti-VEGF application in the study eye and/or any surgical treatment within three months prior to baseline of the study. Patients with uncontrolled glaucoma, aphakia, active intraocular or periocular infection, vitreous hemorrhage, iris neovascularization, retinal detachment and/or presence of uncontrolled systemic disease were also excluded from the study. Further, allergy or hypersensitivity to either fluorescein, indocyanine green or the study drug aflibercept were exclusion criteria.

At each study visit, a complete ophthalmic examination including 4-m ETDRS BCVA assessment, slit-lamp examination, indirect ophthalmoscopy, applanation tonometry and OCT examination was performed. For pupil dilation, patients received tropicamide 1% (Mydriatikum^®^, Agepha; Söding, Austria) eye drops. Central retinal thickness (CRT) was assessed, using a Heidelberg Spectralis SD-OCT^®^ (Heidelberg Engineering, Dossenheim, Germany, software version 1.5.2.0) volume scan (20x20°, 49 frames, high resolution) at all study visits. In addition, a Dynamic Vessel Analyzer (DVA, IMEDOS, Jena, Germany) for retinal vessel diameter and oxygen saturation measurements, and flicker response were performed at baseline (BSL) and in three-monthly intervals. Fluorescein and indocyanine green angiography for assessing the different lesion types and status of the retina (e.g. leakage due to macular edema) were performed at baseline, and at 3, 6, 9 and 12 month follow-up visits.

The Dynamic Vessel Analyzer (DVA; IMEDOS, Jena, Germany) is a non-contact device for measuring the arterial and venous oxygen saturation, vessel diameters and flicker response. Measurements of the temporal retinal arteries and veins between 1 and 2 disc diameters from the margin of the optic disc were used for oxygenation, diameter and flicker response evaluation [15]. The fundus images are done with two wavelengths (610nm and 548nm) using a fundus camera (FF450, Carl Zeiss Meditec AG, Jena Germany). The two wavelengths are used to differ oxygenated from deoxygenated vessels due to the different light absorption levels of hemoglobin. The retinal vessel diameter is automatically detected by vessel analyzing software using adaptive algorithms to delineate vessel borders. The DVA measures the retinal vessel dilatation in response to diffuse luminance flicker. The mean diameter of the arterial and venous vessels is obtained. The responses are represented as an average increase in the vessel diameter in response to the flickering light during the measurements. It shows the percentage increase relative to the baseline diameter size. For evaluation of the flicker response, a baseline measurement was taken for 60 seconds followed by a flicker light stimulation of 30 seconds and 60 seconds of post-flicker relaxation. The frequency of flicker light stimulation was performed at 12.5Hz [15]. All measurements of the arterial and venous oxygen saturation as well as vessel diameters and flicker response were done before administration of the anti-VEGF drug to avoid bias from short-term effects.

### Outcome variables and statistical analysis

Our aim was to detect a mean change in total arterial oxygen saturation of 0.5 standard deviations. Using a paired t-test with a 5% two-sided significance level a sample size of 44 patients was needed to show this effect with a power of 90%. Considering an estimated drop-out rate of roughly 10% the total sample size was determined as 50. Statistical analysis was performed using SPSS (version 20 for windows; SPSS, Inc., Chicago, IL) and R version 3.4.3. Categorical data is presented in frequency and percentage, whereas nominal variables after checking for normal distribution are expressed as mean ± standard deviation. A p-value <0.05 was considered as statistical significant. All analyses were performed at the Department of Medical Statistics at the Medical University of Vienna.

The primary outcome measure was the arterial oxygenation over time. Paired t-tests were performed to assess whether arterial oxygenation changed after loading dose (after 3 months) and until last follow-up (after 12 months). The secondary outcome measures included the venous oxygen saturation, arteriovenous ratio, and retinal vessel diameters as well as the flicker response in arterial and venous retinal vessels. Again, paired t-tests were performed to analyze the effect of intravitreal aflibercept treatment of the total study population after loading dose (after 3 months) and the responses of all parameters at the last follow-up (after 12 months). Further, correlation of oxygen saturation, flicker response and vessel diameter with functional (VA) and morphological (CRT) outcomes were calculated using Spearman correlation coefficient. Results of the 6 and 9 month visits are only given in a descriptive analysis. No correction for multiple testing was applied, therefore all p-values are of descriptive, hypothesis-generating character.

## Results

Forty-four eyes of 44 patients with a mean age of 78±7 years (mean±standard deviation; range 61 to 89 years) were included in the statistical analysis. At baseline, the mean visual acuity was 65±16 letters and mean central retinal thickness was 531±181μm in the total population. Mean visual acuity showed no statistically significant change with 68±17 letters at last follow-up (p = 0.3; mean estimated difference [confidence interval] -3.15 [-3.30; 9.60]). There was a significant reduction of central retinal thickness to 354±134μm (p<0.01; -196.89 [-256.24; -137.55]) compared to baseline. The patients received a mean amount of 6±2 intravitreal aflibercept injections during the entire study period. Regarding safety assessment, no adverse event occurred in relation to the study drug. Some part of the study data is presented in an earlier publication [16].

See Table 1 for more detailed information on the total population and the subgroups.

**Table 1:** Detailed information on the demographic, functional and morphological characteristics at baseline, month 3 and month 12 for the total population and the subgroups.

-	Total population	PCV^a^	hCNV^b^	RAP^c^	PED^d^
**Number of eyes**	44	12	12	9	11
**Age (in years, mean ± standard deviation)**	78 ± 7	79 ± 8	77 ± 8	80 ± 6	75 ± 5
**Sex, male number (%)**	11 (25%)	3 (25%)	4 (33%)	1 (11%)	3 (27%)
**mean number of injections ±standard deviation**	6 ± 2	5 ± 2	6 ± 2	7 ± 2	6 ± 2
**Visual acuity (in letters, mean ± standard deviation, 95% confidence interval)**					
**Baseline**	65 ± 16 (60, 70)	67 ± 16 (56, 77)	55 ± 21 (41, 69)	64 ± 16 (56, 73)	74 ± 7 (69, 79)
**Month 3**	71 ± 15 (66, 75)	72 ± 11 (66, 81)	61 ± 19 (52, 76)	72 ± 15 (60, 85)	75 ± 9 (69, 81)
**Month 12**	68 ± 17 (64, 74)	66 ± 16 (60, 80)	69 ± 17 (54, 78)	68 ± 12 (61, 82)	69 ± 18 (57, 83)
**Central retinal thickness (in μm, mean ± standard deviation, 95% confidence interval)**					
**Baseline**	531 ± 181 (475, 587)	523 ± 251 (354, 691)	497 ± 171 (388, 606)	573 ± 132 (472, 675)	541 ± 158 (435, 647)
**Month 3**	314 ± 94 (284, 343)	261 ± 32 (238, 284)	300 ± 77 (249, 352)	274 ± 40 (241, 307)	413 ± 106 (338, 488)
**Month 12**	354 ± 134 (304, 384)	310 ± 91 (239, 356)	323 ± 75 (265, 366)	357 ± 173 (213, 482)	422 ± 150 (317, 519)

^a^polypoidal choroidal vasculopathy.

^b^hemorrhagic choroidal neovascularization (hCNV).

^c^retinal angiomatous proliferation (RAP).

^d^pigment epithelium detachment (PED).

### Oxygenation and diameter of retinal vessels

The total arterial oxygen saturation was 94.01±2.14% at baseline, 93.94±2.88% at month 3, 93.85±2.74% at month 6, and 95.48±1.90% at month 12 (Analysis BSL to month 3: p = 0.4, -0.38 [-1.24; 0.48] and BSL to month 12: p = 0.1; 1.29 [-0.34; 2.91]). Venous oxygenation was 78.49±6.93% at baseline, 80.94±7.71% at month 3, 78.41±7.44% at month 6 and 80.56±7.33% at month 12 (Analysis BSL to month 3: p = 0.7; -0.43 [-2.72; 1.86] and BSL to month 12: p = 0.5; 1.07 [-2.10; 4.24]).

The arteriovenous (AV) ratio did no change significantly over the observed period (baseline: 0.78±0.11%, month3: 0.77±0.09%; month 6: 0.80±0.09%; month 12: 0.79±0.10%; Analysis BSL-month 3: p = 0.9; -0.00 [-0.03; 0.02] and BSL-month 12: p = 0.7; 0.01 [-0.03; 0.04]).

Regarding the course of arterial retinal vessel diameter, a mean of 94.37±21.84μm was measured at baseline did not change significantly at 96.57±16.48μm at month 3 (p = 0.5, 2.30 [-5.00; 9.59], 93.30±18.63μm at month 6 and 94.83±17.82μm at month 12 (p = 0.5, 2.74 [-5.29; 10.77]). A mean of 131.13±18.98μm venous vessel diameter was observed at baseline, 132.38±15.31μm a month 3, 130.28±18.50μm at month 6 and 129.55±16.10μm at month 12 (p = 0.8 0.59 [-3.17; 4.34] month 3 in comparison to baseline; p = 0.9 0.12 [-4.75; 4.99] month 12 in comparison to baseline).

**Fig 2:**
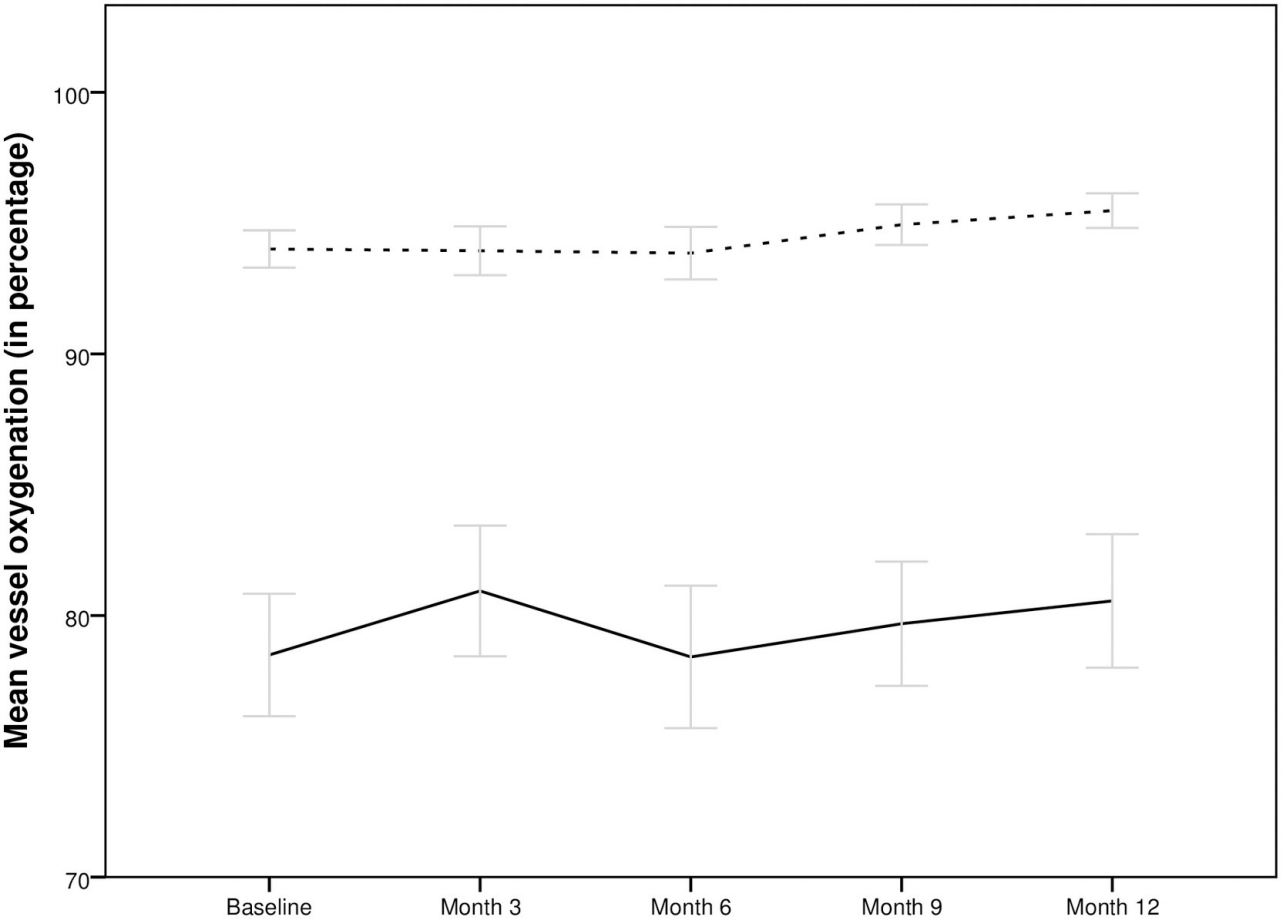
Course of arterial (dotted line) and venous (continuous line) vessel oxygen saturation of the total population during the treatment with aflibercept. No significant change was observed over the 12 months period.

**Fig 3:**
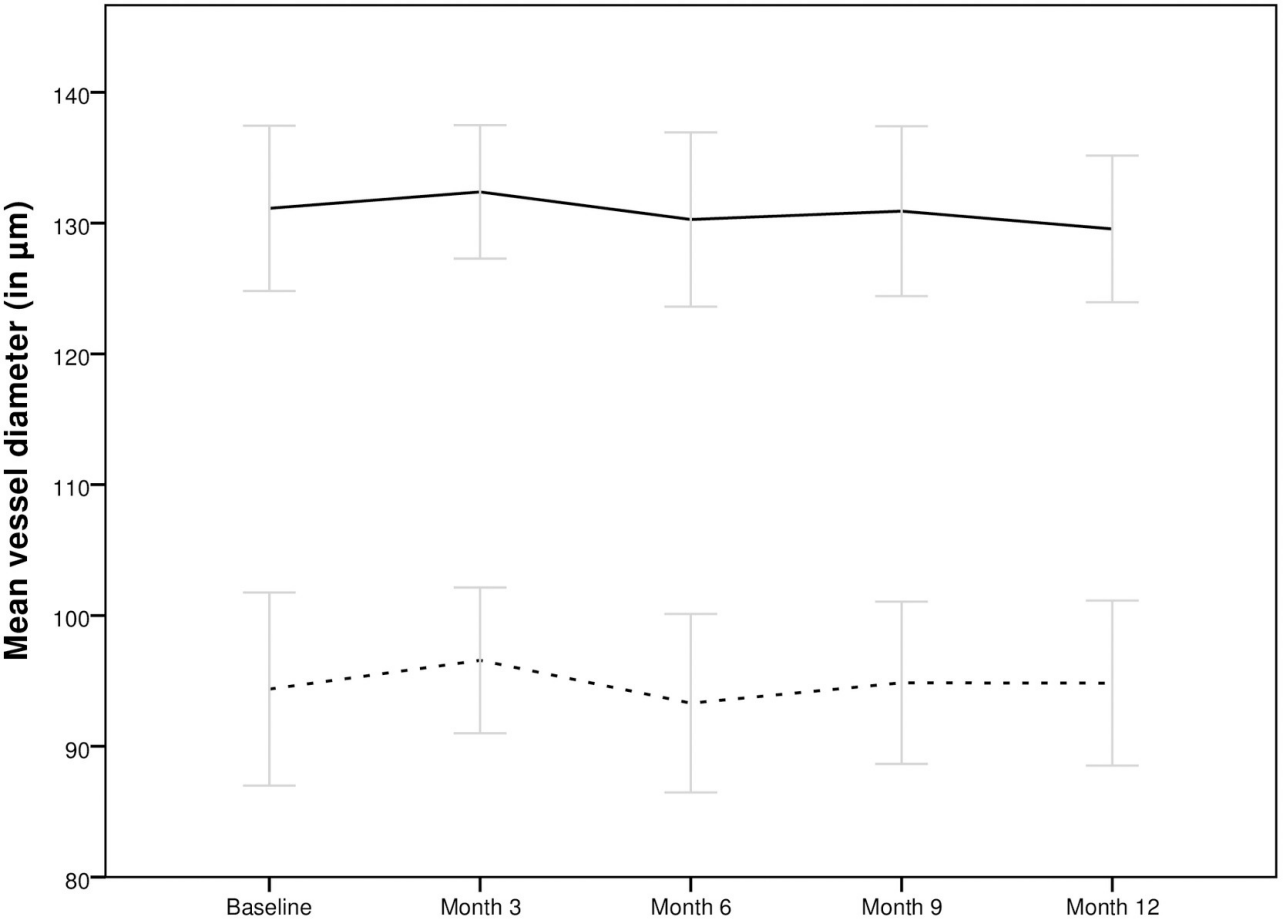
Course of arterial (dotted line) and venous (continuous line) vessel diameter of the total population during the treatment with aflibercept. No significant change was observed over the 12 months period.

Figs 2 and 3 show the course of vessel diameter and vessel oxygenation of the total study population during the observational period and Table 2 shows detailed information of vessel parameters of the subgroups during follow-up.

**Table 2:** Detailed information of the arteriovenous oxygen difference, retinal oxygen saturation and vessel diameter for the subgroups during the observational period.

	PCV^a^ (n = 12)	hCNV^b^ (n = 12)	RAP^c^ (n = 9)	PED^d^ (n = 11)
Arterial oxygen saturation (mean±standard deviation and 95% confidence interval)				
Baseline	95.00±1.68% (94; 95)	93.26±1.82% (92; 96)	93.42±.20% (92; 96)	94.37±2.39% (92; 96)
Month 3	94.22±4.77% (91; 97)	93±69±2.12% (92; 95)	93.49±1.18% (92; 94)	94.29±1.98% (94; 96)
Month 12	95.24±2.77% (93; 98)	95.09±1.57% (94; 96)	94.98±0.86% (94; 96)	96.41±1.55% (95; 98)
Venous oxygen saturation (mean±standard deviation and 95% confidence interval)				
Baseline	82.46±4.06% (97; 86)	76.24±7.32% (71; 82)	72.75±6.59% (67; 69)	81.68±4.27% (78; 85)
Month 3	86.28±6.01% (79; 90)	78.93±8.31% (74; 86)	78.26±8.01% (70; 83)	80.09±5.65% (78; 86)
Month 12	83.64±6.39% (79; 92)	80.77±9.14% (73; 88)	76.07±5.48% (71; 81)	81.49±5.59% (77; 86)
Arteriovenous oxygen difference (mean±standard deviation and 95% confidence interval)				
Baseline	0.81±0.09% (0.8; 0.9)	0.78±0.06% (0.7; 0.8)	0.77±0.14% (0.7; 0.9)	0.74±0.12% (0.6; 0.8)
Month 3	0.73±0.11% (0.7; 0.8)	0.81±0.08% (0.7; 0.9)	0.79±0.05% (0.7; 0.9)	0.75±0.09% (0.7; 0.8)
Month 12	0.82±0.13% (0.7; 0.9)	0.80±0.10% (0.7; 0.9)	0.80±0.08% (0.7; 0.9)	0.75±0.09% (0.7; 0.8)
Arterial vessel diameter (in μm) (mean±standard deviation and 95% confidence interval)				
Baseline	98.29±20.26 (83; 114)	99.17±25.05 (82; 117)	94.23±17.28 (77; 111)	84.26±18.92 (69; 100)
Month 3	88.47±15.81 (83; 114)	90.83±16.68 (92; 110)	98.95±16.28 (77; 113)	107.69±8.36 (82; 100)
Month 12	93.60±25.75 (71; 117)	101.04±15.88 (88; 113)	91.82±15.95 (76; 108)	92.33±9.78 (89;101)
Venous vessel diameter (in μm) (mean±standard deviation and 95% confidence interval)				
Baseline	130.78±13.85 (120; 141)	132.51±27.10 (113; 152)	132.38±15.94 (118; 147)	128.72±13.25 (118; 140)
Month 3	132.64±7.25 (122; 148)	126.91±14.05 (117; 142)	130.34±17.75 (119; 146)	139.46±16.68 (124; 141)
Month 12	122.14±15.70 (108; 136)	130.42±18.40 (115; 145)	131.49±12.91 (120; 143)	133.16±14.54 (122;144)
Arterial flicker response (mean±standard deviation and 95% confidence interval)				
Baseline	+0.63±2.78% (-1.1; 2.3)	+2.15±6.17% (-2.4; -0.02)	+2.86±5.83% (-9.3; 6.1)	-0.22±3.61% (-5.1; 1.3)
Month 3	+3.83±4.31% (-0.5; 4.7)	+1.10±3.39% (-2.4; 4.8)	+2.52±6.01% (2.9; 0.3)	+3.34±8.45% (-6; 8.4)
Month 12	+1.40±2.38% (-3.5; 3)	+3.45±5.75% (-3; 4.4)	+2.64±4.45% (-3.4; 0.8)	+3.39±.32% (-1.4; 1.4)
Venous flicker response (mean±standard deviation and 95% confidence interval)				
Baseline	+5.32±2.89% (-0.2; 1.4)	+6.16±4.53% (-2.3; 1.1)	+2.08±5.73% (3.7; 3.0)	+4.00±3.35% (-1.8; 2.1)
Month 3	+6.05±4.20% (0.4; 5.3)	+3.18±1.16% (-0.7; 3.3)	+3.18±2.16% (-1.1; 0.9)	+3.85±4.53% (-0.9; 6.5)
Month 12	+4.37±1.72% (2.5; 4)	+2.42±7.04% (-2; 5)	+6.34±2.73% (-1; 2)	+4.03±4.53% (-0.9; 0.6)

Mean±standard deviation and 95% confidence interval of all values is provided.

^a^polypoidal choroidal vasculopathy.

^b^hemorrhagic choroidal neovascularization (hCNV).

^c^retinal angiomatous proliferation (RAP).

^d^pigment epithelium detachment (PED).

### Flicker response

Stimulation with flicker light showed no significant response in arterial or venous retinal vessels during the study period. The arterial flicker response in the total population was +1.24±4.93% at baseline. At month 3, the mean change increased to +2.70±5.95% (p = 0.5; 1.43 [-2.54; 5.41]), +2.80±4.83% at month 12 (p = 0.04; 1.47 [-3.89; 6.82). At baseline, change of venous flicker response was +4.52±4.45%, +4.13±3.65% at month 3 (p = 0.4; 5.18 [1.73; 8.63]), and +4.27±5.75% at month 12, respectively (p = 0.5; -0.08 [-5.10; 4.95]).

**Fig 4:**
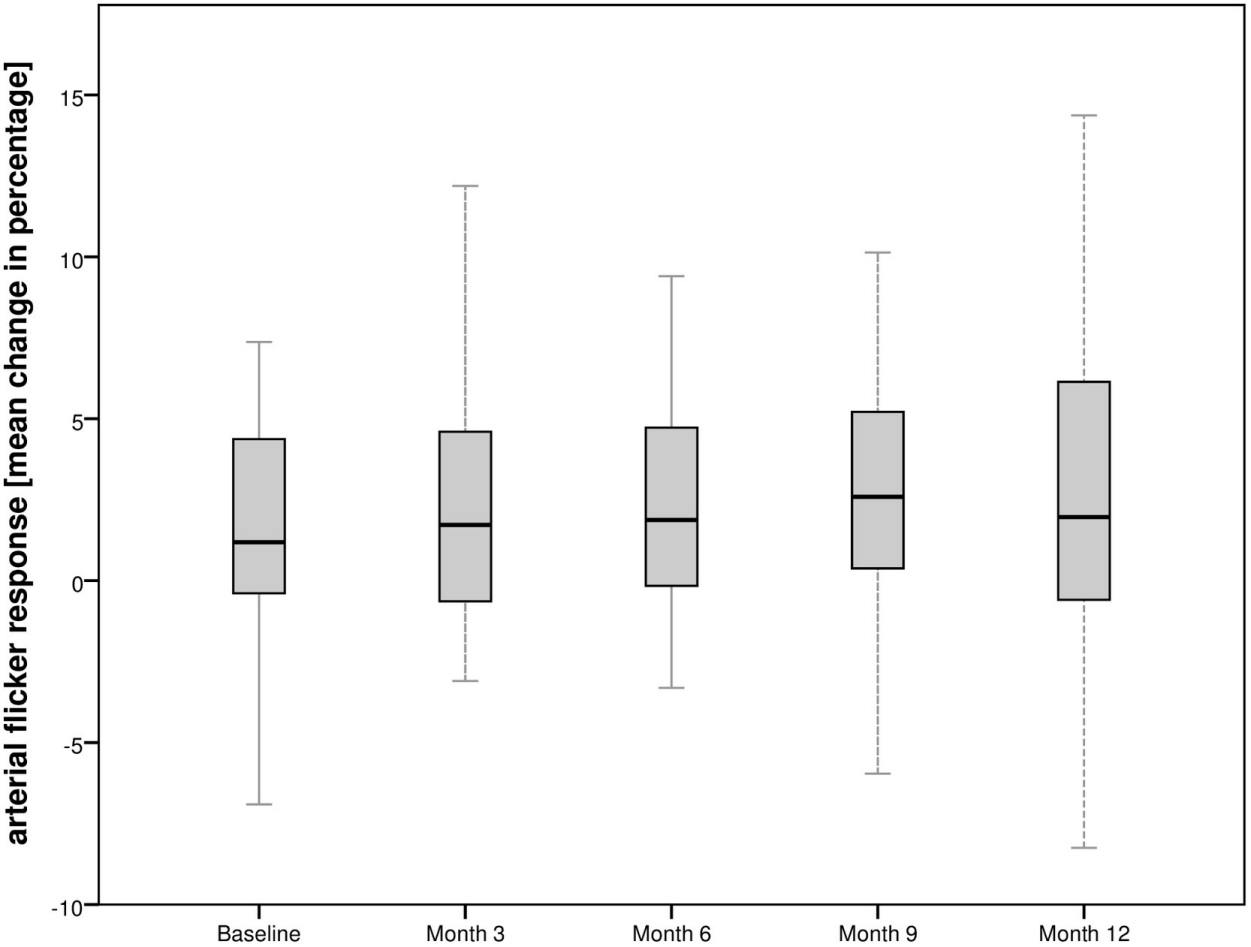
Course of arterial flicker response for the total population undergoing intravitreal aflibercept injections over the observational period.

**Fig 5:**
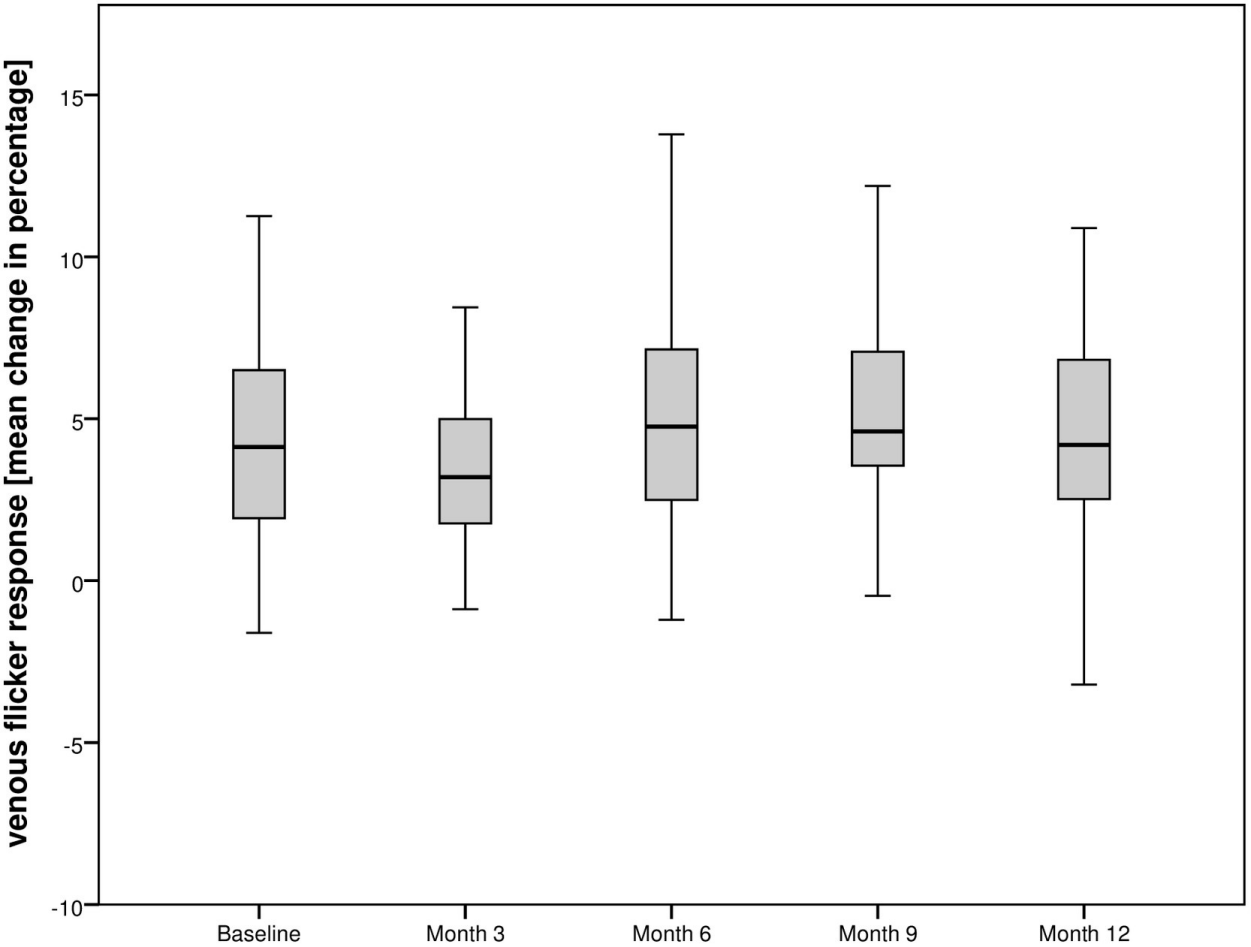
Course of venous flicker response of the total population undergoing intravitreal aflibercept injections over the observational period.

Figs 4 and 5 show the change of arterial and venous flicker response of the total population over the observational period and Table 2 shows the flicker response of the subgroups during the observational period.

### Correlations with functional and anatomical parameter

The main outcome parameter, arterial oxygenation, showed no significant correlation to visual acuity (spearman correlation coefficient: baseline: r = -0.19, p = 0.57; month 3: r = -0.20, p = 0.06; month 12: r = -0.27, p = 0.70) and the central retinal thickness (baseline: r = 0.26, p = 0.50; month 3: r = -0.07, p = 0.06; month 12: r = 0.08, p = 0.70). No significant correlations were found at any time point for the secondary outcome measures, venous oxygen saturation and retinal vessel diameter to the visual and anatomic outcome parameters, except venous oxygen saturation which showed a moderate negative correlation with visual outcome at month 12 (r = -0,54; p<0.01).

## Discussion

In this prospective study, we investigated the effect of intravitreal aflibercept on the hemodynamic parameters arterial and venous oxygen saturation, vessel diameter and flicker response in subtypes of exudative AMD, in particular PCV, type 3 MNV, predominantly PED and hCNV lesions. For anti-VEGF therapy, including ranibizumab and bevacizumab, vasoconstriction of retinal vessels was shown before [17–19]. However, no data exist regarding the course of hemodynamic parameters during aflibercept treatment and the course in subtypes of neovascular AMD. In clinical trials, mainly type I and type II lesions are evaluated excluding other subtypes of neovascular AMD. However, the course of disease is known to differ for type I or II lesions in comparison to subtypes of AMD as they are known to have a worse treatment response and poor prognosis [7–11]. To our knowledge, this is the first study evaluating the oxygen saturation, vessel diameter and flicker response in these subtypes of neovascular AMD during aflibercept treatment. The use of anti-VEGF agents has been widely expanded and the knowledge about their hemodynamic consequences in the retina is important for treatment response and management of the patients.

Vascular endothelial growth factor (VEGF) has several effects on the retinal vasculature by increasing vascular permeability, stabilizing vascular endothelial cells and is a potent vasodilatator acting via nitric oxide. Anti-VEGF agents including ranibizumab and bevacizumab were shown to reduce VEGF levels thereby causing vasoconstriction to the retinal vessels. However, the vasoconstrictive effect was also hypothesized to cause further ischemia which is associated with substantial visual loss [20]. In addition, studies [21, 22] showed that anti-VEGF injections may be associated with disturbance in retinal blood flow.

In our analysis [23], arterial oxygenation of 94.01% at baseline was comparable to other studies on AMD. No significant change of arterial oxygenation was found following aflibercept loading dose and at the end of the study in the total population, as well as in the subgroups when looking at the absolute measurements. Similar results were found for the venous oxygenation and arteriovenous ratio. These results suggest that aflibercept does not affect the oxygen supply and therefore, does not impair tissue oxygenation.

Papadopoulou et al. [19] described a reduction of arteriolar diameter following three monthly ranibizumab injections in nAMD, whereas Micieli et al. [18] observed a reduction in arterial diameter, blood velocity and blood flow. In nAMD patients, studies included mainly classic, occult and minimally classic lesions and for those lesions a significant decrease of the retinal arteriolar diameter was observed after each intravitreal injection. In addition, those studies evaluated patients 30 days after the intravitreal injection. In our analysis we followed the patients until 12 months and measured the effect after repeated treatments. Furthermore, literature regarding aflibercept and its influence on ocular hemodynamics is sparse. Information about subtypes of neovascular AMD is missing. In addition, a vasoconstrictive effect was described for ranibizumab and bevacizumab, though not yet for aflibercept. For both vessel compartments, arterial or venous, we observed no change in the total population, neither after the three-month loading dose nor at the last follow-up. But in the subtypes of AMD we observed a decrease of the arterial diameter in PCV and hemorrhagic CNV after loading dose. However, these results were not reproducible at the last visit. No such changes were seen in the other two forms, type 3 MNV and PED. In our analysis initial loading dose of three injections in monthly intervals were administered followed by retreatment in bi-monthly intervals. However, there are patients in need of more frequent injections and knowledge about the effect of higher amount of anti-VEGF administration on the vascular parameters is important and should be addressed in future studies.

Flickering light stimulation has been proven to cause retinal vessel dilation in humans, since ganglion cells are activated and release local vasodilating factors. Several studies [24–26] examined the effect of different drugs or blood concentrations on the flicker response. Garhofer et al. [27] evaluated the blood lactate levels and observed a reduced response if lactate was elevated. A similar response was found for excessive hyperglycemia (>300mg/dl) causing a reduced flicker response [28]. Alterations of flicker-induced vasodilation were evaluated in several pathologies such as early open angle glaucoma, diabetes and AMD [29, 30]. In our study, we observed no change in flicker response during the follow-up examinations at month three and twelve for neither arterial nor venous retinal vessels in subtypes of neovascular AMD. Although in some studies [31, 32] blood flow regulation was found to be impaired in AMD and flicker-induced vasodilation to be altered. Compared to our study, we did not include advanced AMD but special subtypes of AMD. Further, anti-VEGF treatment was performed using aflibercept injections not bevacizumab, which was found to significantly affect ocular hemodynamic parameters [1].

When interpreting the results of the present study, limitations need to be mentioned. A limitation of this study was that no healthy controls were included and therefore, did not allow a comparison to AMD patients with respect to normalization or worsening of the study parameters. Moreover, it is impossible nowadays to not treat patients with AMD and just to observe the natural course of the disease in relation to our outcomes. Therefore, we cannot draw conclusions about the effect of the disease during the course of the study. Furthermore, OCTA technology allows the visualization of very small retinal vessel and allows differentiation between the superficial and deep vascular plexus. With OCTA additional information about the vessel density and foveal avascular zone can be obtained. So, future studies should also evaluate the differences in retinal vessels using OCTA in the different subforms of neovascular age-related macular degeneration. However, this study shows the course of the retinal oxygen saturation, vessel diameters and flicker response in a cohort of patients with different subtypes of age-related maculopathy during aflibercept treatment over a 12 month period. But, due to the small population size for each subtype, no statistical analysis was performed on the response to aflibercept during the study and additional studies including, for instance only one subtype, are necessary to provide further information.

Another limitation is that the ocular blood flow was not evaluated in study population. These results would have allowed a better insight in the changes of vasculature under aflibercept treatment and should, therefore, be performed in future.

In conclusion, no change of the arterial oxygen saturation was found in specific subtypes of AMD, PCV, hCNV, type 3 MNV and PED following intravitreal aflibercept treatment over the 12-month period. Furthermore, venous oxygenation, retinal vessel diameters and flicker response remained relatively constant indicating no effect of aflibercept on hemodynamic parameters.

## Supporting information

S1 Checklist TREND statement checklist. (PDF)

S1 Dataset Minimal data set. (XLSX)

S1 File (PDF)
