# Supplementary material for: Retinal oxygen saturation, vessel diameter and flicker response in eyes with specific subtypes of neovascular age-related macular degeneration during aflibercept treatment
Source: PLoS One. 2022 Jul 12;17(7):e0271166. doi: 10.1371/journal.pone.0271166 (PMC9275690; doi:10.1371/journal.pone.0271166)
Supplement: S1 File — (PDF) [file pone.0271166.s003.pdf]

Department of Ophthalmology and Optometry
Medical University of Vienna

Allgemeines Krankenhaus
Währinger-Gürtel 18-20
1090 Vienna
Austria
Tel.: + 43 1 40400 7940
Fax. + 43 1 40400 7912
e-mail: ophthalmology@meduniwien.ac.at

MICROSTRUCTURAL CHANGES BY INTRAVITREAL AFLIBERCEPT TREATMENT IN RAP-LESIONS, PED, HEMORRHAGIC CNV AND PCV

Study Synopsis

Version 3.0

(04.09.2014)

Stefan Sacu[1], Katharina Eibenberger[1], Sandra Rezar[1], Wolf Bühl[1], Günther Weigert[1], Leopold Schmetterer[2], Ursula Schmidt-Erfurth[1]

Medical University of Vienna
1 Department of Ophthalmology
2 Clinical Pharmacology

Principal Investigator:
Stefan Sacu, MD
Department of Ophthalmology and Optometry
Medical University of Vienna
Währinger Gürtel 18-20, 1090 Vienna
phone:+431-40400-7962, fax: -7932
e-mail: stefan.sacu@meduniwien.ac.at

Study coordinator:
Katharina Eibenberger, MD
Department of Ophthalmology and Optometry
Medical University of Vienna
Währinger Gürtel 18-20, 1090 Vienna
e-mail: katharina.eibenberger@meduniwien.ac.at

SYNOPSIS

TITEL: INTRAVITREAL AFLIBERCEPT TREATMENT IN RAP-LESIONS, PED, HEMORRHAGIC CNV AND PCV

SHORT TITLE: Aflibercept treatment for RAP, PED, hemorrhagic CNV and PCV

MAIN INVESTIGATOR: Assoc. Prof. PD Dr. Stefan Sacu,
Department of Ophthalmology, Medical University Vienna, Vienna General Hospital, Währinger Gürtel 18-20, A-1090 Wien, Tel. +43-1-40400-7900,
Fax. +43-1-40400-7932; email: stefan.sacu@meduniwien.ac.at

CO-INVESTIGATOR Katharina Eibenberger MD, Sandra Rezar MD, Wolf Bühl MD, Günther Weigert MD, Leopold Schmetterer PhD, Ursula Schmidt-Erfurth MD
Department of Ophthalmology and Optometry, Medical University of Vienna
Währinger Gürtel 18-20, A-1090 Vienna

SPONSOR Medical University Vienna
Spitalgasse 23; 1090 Vienna

BACKGROUND: Choroidal neovascularization (CNV) is a hallmark sign of wet age-related macular degeneration (AMD). There are various type of wet AMD with different frequency and prognosis. RAP lesions, PED, hemorrhagic CNV and PCV are subforms of exsudative maculopathy with an aggressive course and poor clinical results. Intravitreal anti-VEGF treatment is widely used and accepted treatment modality in these cases too. A novel anti-VEGF agent aflibercept is approved for the treatment of neovascular AMD in European Union in November 2012. Aflibercept is a recombinant fusion protein consisting of portions of human VEGF receptors 1 and 2, and acts as a soluble decoy receptor that binds VEGF-A and placental growth factor (PlGF). There is only sparse knowledge concerning the clinical outcomes of intravitreal aflibercebt (Eylea, Bayer) in these subforms. With an additional inhibition effect on PlGF, aflibercept may show better results in this aggressive subforms of exsudative AMD. Hence the present study is conducted to assess the clinical outcomes of intravitreal aflibercept in eyes with RAP lesions, PED, hemorrhagic CNV and PCV. Furthermore, the influence of intravitreal

afliberfcept on retinal pigment epithelial changes and its association with vision outcome will be evaluated.

AIM: The purpose of the study is to assess the evaluate of intravitreal aflibercept (Eylea®, Bayer) on clinical outcomes and retinal pigment epithelium in eyes with RAP-lesions, PED, hemorrhagic CNV or PCV.

STUDY DESGIN: Prospective non-randomized clinical trial

STUDY POPULATION 50 patients with RAP-lesions, PED, hemorrhagic CNV or polypoidal choroidal vasculopathy (PCV).

METHODS Prospective non randomized clinical study.
Patients will be examined in monthly interval during follow-up time of 12 months after initial treatment.

EXAMINATIONS
Best-corrected visual acuity (BCVA) using ETDRS charts at 4m distance
Standard ophthalmic examinations including funduscopy and applanation tonometry
Retinal Vessel Analyzer (RVA)
Color Doppler Imaging (CDI)
Laser Doppler Velocity (LDV)
Oxymap T1 Retinal Oximeter
Optical coherence tomography (OCT) using Spectralis-OCT,
Polarization sensitive OCT (POL-OCT)
Macula pigment optical density as measured with optical reflectometry (MPOD)
Fluorescein angiography and indocyaningreen angiography (FLA/ICG)

At baseline, three, six, nine and twelve months all of the above mentioned examinations will be performed.
At any other monthly visit following examinations will be performed: BCVA, Spectralis-OCT and standard ophthalmic examination,

STUDY MEDICATION Aflibercept (Eylea ®, Bayer)
Study patients will initially receive three injections in monthly intervals (loading dose) and then treatment will be administered in bimonthly intervals according to the approved label. Treatment will be stopped upon improvement

or stabilization of sub-/intraretinal fluid or subretinal hemorrhages during 3 consecutive follow-up visits.

OUTCOME MEASURES:

Main Outcomes:

alteration in retinal pigment epithelium as assessed with POL-OCT
Oxygen saturation of retinal vessels

Additional outcome variables:

best-corrected visual acuity
anatomic changes in the macula as assessed with Spectralis-OCT
Retinal vessel diameters
retrobulbar flow velocities
retinal blood flow
Macula pigment optical density
chorioretinal perfusion (ICG)
perfusion of the neovascular net (FLA and ICG)

Safety variables:

systolic/diastolic blood pressure (non-invasive),
pulse rate
Other adverse events.

RISK/BENEFIT ASSESSMENT: Intravitreal application of aflibercept has been approved in European Union in 2012 and is used for the treatment of exsudative age-related maculopathy worldwide. This study aims to evaluate the therapeutic effect of intravitreal aflibercept in patients with aggressive subforms of exsudative age-related maculopathy with focus on the microstructural changes. The results will reveal important information about treatment effectiveness of subtypes of exsudative age-related maculopathy in real-life. All imaging procedures used in this study are non-invasive and painless. The risk/benefit ratio is therefore acceptable.

Table of Contents

1. INTRODUCTION
1.1. Background
1.2. Rationale of the Study
1.3. Risk/benefit assessment

2. STUDY OBJECTIVES

3. INVESTIGATIONAL PLAN
3.1. Design
3.2. Selection of study population
3.2.1. Number of subjects
3.2.2. Inclusion and exclusion criteria
3.2.3. Outcome variables
3.3. Treating the patient
3.3.1 Study medication
3.3.2. Intravitreal aflibercept application
3.3.3. Drug Accountability
3.3.4. Risks and Precaution of intravitreal Aflibercept application

4. METHODS
4.1. Visual Acuity
4.2. Standard ophthalmic examination
4.3. Fluorescein/ICG angiographies
4.4. Optical coherence tomography (OCT)
4.4.1 Spectral Domain OCT
4.4.2 POL-OCT
4.5. Macular pigment optical density (MPOD)
4.6. Retinal Vessel Analyzer (RVA)
4.7. Laser Doppler Velocity (LDV)
4.7.1. Retinal blood flow
4.8. Color Doppler Imaging
4.9. Oxymap T1 Retinal Oximeter
4.10. Adverse events
4.10.1. Definition of serious and non-serious adverse events
4.10.2. Reporting/Documentation of AE
4.10.3. Assessment of severity

4.10.4. Withdrawal rules and premature termination of the study
4.11. Data handling procedures
4.12. Biometric methods
4.12.1. Biometric methods – statistic planning and outcome variables
4.12.2. Biometric methods - Adverse events/Safety investigations

5. ETHICAL AND LEGAL ASPECTS
5.1. Informed consent of subject
5.2. Acknowledgment/approval of the study and trial registration
5.3. Insurance
5.4. Confidentiality
5.5. Monitoring

6. DOCUMENTATION AND USE OF STUDY FINDINGS
6.1. Documentation of study findings
6.2. Use if study findings

7. PROTOCOL AMENDMENTS

8. REFERENCES

APPENDICES
Subject information sheet and consent form

1 INTRODUCTION

1.1. Background

Choroidal neovascularization (CNV) is a hallmark sign of wet age-related macular degeneration (AMD). Less frequent subtypes of wet AMD are RAP-lesions, PED, hemorrhagic CNV, and polypoidal choroidal vasculopathy (PCV). (1; 2; 3; 4)

Retinal angiomatous proliferation (RAP) are characterized by small multiple intra-retinal hemorrhages, edema, vascularized pigment epithelial detachments and retinal choroidal anastomosis (RCA). RAP-lesions may precede or follow the development of wet AMD. (2)

Further characteristics of AMD are retinal pigment epithelium detachments (PED) with “waste” products and lipid deposits accumulating in Bruch´s membrane, which can cause CNV. Several studies investigating the underlying pathogenesis propose inflammatory pathways or reduced hydraulic conductivity. Therefore treating PED with intravitreal injections to reduce the area of PED may prevent CNV and preserve good visual acuity. (3)

Hemorrhagic CNV is characterized by massive macular bleeding on initial presentation. The visual prognosis is worse than for typical CNV (classic and occult) since bleeding aggravates the destruction of photoreceptors and RPE. In addition, there are no standardized therapeutic approaches available. Further studies are necessary to evaluate the efficacy of potential therapeutic options. (1).

Polypoidal choroidal vasculopathies (PCV) were first described as a peculiar hemorrhagic disorder of the macula characterized by recurrent sub-retinal and sub-pigment epithelium bleeding. The inner choroidal vascular network shows pathological aneurysmal bulges. Clinically, it is associated with chronic, multiple, recurrent sero-sanguineous detachments of the retinal pigment epithelium and neurosensory retina with long-term preservation of good vision. (4)

In the large multicenter trials for the approval of anti-VEGF treatment for age-related macular degeneration usually classic and occult lesions were evaluated based on the visual outcome function. (5,6) But there is missing evidence on the functional and morphological outcomes of real-life cases with aggresive subtypes of exsudative maculopathies. This study investigates the treatment effect of aflibercept on function, retinal morphology assessed with spectral domain OCT. Additionally changes in retinal pigment epithelium evaluated in POL-OCT and oxygen saturation of the retinal vessels under intravitreal aflibercebt were assesed.

1.2. Rationale of the Study

With an inhibition effect on VEGF and PlGF, aflibercept may show better results in aggressive subforms of exsudative AMD with poor outcomes. Hence the present study is conducted to evaluate the clinical outcomes of intravitreal aflibercept in eyes with RAP

lesions, PED, hemorrhagic CNV and PCV. Furthermore, the influence of intravitreal afliberfcept on retinal pigmentepithelial changes and its association with vision outcome will be evaluated.

1.3. Risk/benefit assessment

Intraretinal application of aflibercept has been approved in European Union in 2012 and is used for the treatment of exsudative age-related maculopathy worldwide. This study aims to evaluate the therapeutic effect of intravitreal aflibercept in patients in exsudative age-related maculopathy with focus on the microstructural changes. The results will reveal important information about treatment effectiveness of subtypes of exsudative age-related maculopathy. All imaging procedures used in this study are non-invasive and painless. The risk/benefit ratio is therefore acceptable

2 STUDY OBJECTIVES

To assess the treatment effect of intravitreal aflibercept in patients with retinal angiomatous proliferation, pigment epithelium detachment, hemorrhagic CNV and polypoidal choroidal vasculopathy on pigment epithelium changes in POL-OCT, oxygen saturation, visual acuity, OCT-morphology, ocular blood flow and macula pigment optical density.

3 INVESTIGATIONAL PLAN

3.1. Design

This is a prospective, non-randomized, open-lable phase IV clinical study.
Patient eligibility based on visual acuity, ophthalmic exam and fluorescein and indocyanine green angiography will be evaluated at the baseline visit.
Overall duration of the study averages out to 2 years, duration for study patient is set for one year.

3.2. Selection of study population

The participants will be selected by the Department of Ophthalmology and Optometry, Medical University of Vienna

Study visits and examinations:

	Baseline	M 1	M 2	M 3	M 4	M 5	M 6	M 7	M 8	M 9	M 10	M 11	M 12
BCVA	X	X	x	x	x	x	x	x	x	x	x	x	x
OCT	X	X	x	x	x	x	x	x	x	x	x	x	x
Oxymap T1	X			x			x			x			x
RVA	X			x			x			x			x
CDI	X			x			x			x			x
LDV	X			x			x			x			x
POL-OCT	X			x			x			x			x
Ophthalmic Examination	X	X	x	x	x	x	x	x	x	x	x	X	x
MPOD	X			x			x			x			x
FLA/ICGA	X			x			x			x			x
Adverse Events	X	X	x	x	x	x	x	x	x	x	x	X	x

3.2.1. Number of subjects

Fifty patients will be enrolled in the study. The population will consist patients over 50 years of age with treatment naïve, RAP-lesions, PED, hemorrhagic CNV and polypoidal choroidal vasculopathy. If both eyes are eligible, the one with the worse visual acuity will be selected for treatment and study unless, based on medical reasons, the investigator deems the other eye the more appropriate candidate for treatment and study.
Eligibility will be assessed at the first visit based on inclusion/exclusion criteria and monitored during the study period.

3.2.2. Inclusion and exclusion criteria

Inclusion criteria

Adults ≥ 50 years

Treatment naïve eyes with following subtypes of exsudative maculopathies will be included:

- Retinal angiomatous proliferation lesions (RAP)
- Pigment epithelium detachment (PED)

- Hemorrhagic CNV (if the size of hemorrhage is either ＞50% of the lesion area or ＞1 disk area in size))
- Polypoidal choroidal vasculopathy (PCV)

Patients who have a BCVA score better than 20/400 in the study eye using ETDRS

Willingness and able to comply with clinic visits and study-related procedures

Proved a signed informed consent form

The patient can take his medicine in the prescribed manner. The prescribed drugs do not constitute an exclusion criteria.

<u>Exclusion criteria</u>

Any prior treatment for exsudative maculopathy including photodynamic therapy and intravitreal anti-VEGF application in the study eye

Any surgical treatment of the eye within 3 months prior to baseline in the study eye

History of glaucoma filtration surgery, corneal transplant surgery or extracapsular extraction of cataract with phacoemulsification within six months preceding Visit 1, or a history of post-operative complications within the last 12 months preceding Visit 1 in the study eye (uveitis, cyclitis etc.)

History of uncontrolled glaucoma in the study eye (defined as intraocular pressure ≥ 25 mmHg despite treatment with anti-glaucoma mediation)

Aphakia or absence of the posterior capsule in the study eye

Presence of a retinal pigment epithelial tear involving the macula in the study eye.

Any concurrent intraocular condition in the study eye (e.g., cataract or diabetic retinopathy) that, in the opinion of the investigator, could either require medical or surgical intervention during the twelve-month study period to prevent or treat visual loss that might result from that condition

Active intraocular inflammation (grade trace or above) in the study eye.

Active or suspected ocular or periocular infection in the study eye.

Any active infection involving eyeball adnexa

Vitreous hemorrhage or history of rhegmatogenous retinal detachment or macular hole in the study eye

Current iris neovascularization, vitreous hemorrhage, or tractional retinal detachment in the study eye

Evidence of infectious blepharitis, keratitis, scleritis, or conjunctivitis in either eye

Other ocular conditions that require chronic concomitant therapy with systemic or topical ocular corticosteroids. Chronic concomitant therapy is defined as multiple doses taken daily for three or more consecutive days at any time within six months prior to screening or during the course of the study.

Pregnant or breast-feeding women.

Women of childbearing potential with either a positive pregnancy test result or no pregnancy test at baseline are excluded. Postmenopausal women must be amenorrheic for at least 12 months in order not to be considered of child bearing potential.

Sexually active men or women of childbearing potential who are unwilling to practice adequate contraception during the study are excluded. (adequate contraceptive measures include stable use of oral contraceptives or other prescription pharmaceutical contraceptives for 2 or more menstrual cycles prior to screening; intrauterine device [IUD]; bilateral tubal ligation; vasectomy; condom plus contraceptive sponge, foam, or jelly or diaphragm plus contraceptive sponge, foam, or jelly)

Allergy to fluorescein

Hypersensitivity to the active substance aflibercept or to any of the excipients (Polysorbate 20, Sodium dihydrogen phosphate, monohydrate, Disodium hydrogen phosphate, heptahydrate, Sodium chloride, Sucrose)

3.2.3. Outcome variables

<u>Main outcome variables:</u>

alteration in retinal pigment epithelium as assessed with POL-OCT
Oxygen saturation of retinal vessels

<u>Additional outcome variables:</u>

best-corrected visual acuity
anatomic changes in the macula as assessed with Spectralis-OCT
Retinal vessel diameters
retrobulbar flow velocities
retinal blood flow
Macula pigment optical density
chorioretinal perfusion (ICG)
perfusion of the neovascular net (FLA and ICG)

<u>Safety variables:</u>

systolic/diastolic blood pressure (non-invasive),
pulse rate
Other adverse events.

3.3. Treating the patient

3.3.1 Study medication

Study patients will initially receive three injections in monthly intervals (loading dose) and then treatment will be administered in bimonthly intervals according to the approved label. Treatment will be stopped upon improvement or stabilization of sub-/intraretinal fluid or subretinal hemorrhages during 3 consecutive follow-up visits.

3.3.2. Intravitreal aflibercept application

Intravitreal aflibercept treatment will be performed identically under sterile conditions in the surgery room as follows: 0,5ml of 2 mg of commercially available aflibercept (Eylea®; Bayer) will be applied intravitreal through the pars plana using a 30-gauge needle.
Preoperative preparation involves thorough cleansing of the lid, lashes, and periorbital area with an antiseptic and topical anesthesia using Oxybuprocain 0.4% and Lidocain 4% eye drops. Postoperative treatment will involve dexamethasone and gentamicin eye drops 4 times a day for 4 days in total.

3.3.3. Drug Accountability

Pharmaceutical form

Solution for injection as pre-filled syringe. The solution is a clear, colorless to pale yellow and iso-osmotic solution. One solution per blister pack.

Order and Storage

The study drug will be ordered by in-house pharmacy institution. Aflibercept will be stored in a refrigerator (2 ° C - 8 ° C). The pre-filled syringe in its blister pack and in the outer carton to protect from light. Before applying the unopened blister Eylea can be up to 24 hours at room temperature are kept (below 25 ° C). After the blister opening further handling must be carried out under aseptic conditions.

Documentation

Receipt and release of the study drug will be documented eCR-Form.

3.3.4. Risks and Precaution of intravitreal Aflibercept application

Intravitreal aflibercept therapy is considered to be safe. As with every intravitreal form of therapy, the potential for post-injection IOP elevation, endophthalmitis, cataract, hemorrhage, rhegmatogenous retinal detachment or proliferative vitreoretinopathy must be considered. However, all of these side effects have an incidence of less than 1% of all treatments.

After intravitreal injections, visual acuity of patient will be checked (e.g. hand motion, counting fingers) to avoid unacceptable IOP elevations and ensure ocular perfusion-in cases of blocked ocular perfusion (i.e. the patient is unable to recognize hand motion/finger counting), a paracentesis will be performed by the surgeon.

4 METHODS

4.1. Visual Acuity

Best-corrected visual acuity (BCVA) will be assessed during selected study visits using best correction determined from protocol refraction. BCVA measurements will be taken in a sitting position using ETDRS charts at 4 meters distance.

To eliminate bias in measurement of the best-corrected VA, the vision examiners will be masked. Vision examiners must not have access to study patient records and must not elicit historical information from the patient regarding vision. The vision examiners will be given access to prior refraction, but should not have access to any prior VA results. The VA assessors will also be masked to the baseline lesion size.

4.2. Standard ophthalmic examination

A standard ophthalmic examination will be carried out using the indirect stereo ophthalmoscope and the slit lamp for direct stereoscopic fundus biomicroscopy. After assessment of BCVA intraocular pressure (IOP) is measured with an applanation tonometer. Applanation tonometry measures the force required to flatten a small area of the central cornea. It is widely used by ophthalmologists and more accurate than Schiotz tonometry. Pupils will be dilated with eye drops (e.g.tropicamide).

4.3. Fluorescein/ICG angiographies

Investigators will use the digital fluorescein angiogram (HRA 2 or Spectralis) to determine presence or absence of CNV. Investigators may apply the standards of their practice to determine diagnosis by fluorescein/ICG angiographies.

Fluorescein/ICG angiographies will be conducted in conjunction with color fundus photography at selected visits. After pupil dilation and prior to fluorescein dye injection, red-free photographs should be taken of the macula of both eyes.

Next, 5 ml of ICG dye is injected into an arm vein. Photographs of the study eye are taken during the early transit phase, at 1 minute, at 5, 10 and 25 minutes. Next, 5 ml of fluorescein 10% dye is injected into an arm vein. Photographs of the study eye are taken during the early transit phase from 15 to 45 seconds, at 60 to 90 seconds, and at 5 and 10 minutes after completion of the fluorescein dye injection.

4.4. Optical coherence tomography (OCT)

During this study two different OCT devices will be used: Spectralis Heidelberg OCT and the prototype of polarization sensitive OCT (Pol-OCT).

4.4.1 Spectral Domain-OCT

In the last decade, a novel non-invasive optical biomedical imaging technology, called optical coherence tomography (OCT) has been developed. OCT is a technology for ophthalmic diagnosis which can perform non-contact, non-invasive, real time, cross-sectional imaging of a variety of ocular structures. It is analogous to conventional ultrasonic pulse-echo imaging, except that OCT uses infrared light rather than acoustic waves.

<u>Infection risk</u>

Ophthalmologic OCT imaging is performed noncontact so there is minimal risk of infection to the patient. Exposed surfaces near the patient’s eye as well as the chin rest of the instrument are cleaned with alcohol before each patient examined.

<u>Electrical Haze</u>

There are no electrical connections to the patient. Therefore, the risk of electrical contact or shock to the patient is extremely low.

4.4.2 POL-OCT

Patients are evaluated with the prototype of polarization sensitive OCT (PS-OCT) developed by the center of medical physics and biomedical engineering in order to get information about the changes at the level of the retinal pigment epithelium. The PS-OCT is a device which is a functional extension of OCT that provides information beyond backscattered intensity to identify retinal pigment epithelial pathologies. PS-OCT has already be demonstrated to adequately determine changes at the RPE level in various retinal diseases. Patients will be examined by acquiring of three-dimensional images of the size of 6.2 x 6.7 x 3.3mm³ (X x Y x Z) with an axial resolution of 4.5 µm in tissue. The RPE was evaluated for the following changes: RPE-thinning, interrupted RPE, focal atrophy, geographic atrophy, accumulation or exsudates.

4.5. Macular pigment optical density (MPOD)

Spectral fundus reflectance will be measured with a densitometer as described previously (Berendschot et al. 2000). For this purpose measurement of fundus reflectance across the visual spectrum is performed using a rotating wheel containing 14 interference filters. An illumination field of approx. 2 degrees centered at the fovea will be used. To obtain an estimate of MPOD spectral reflectance is measured perpendicular and oblique. Using an optic model of fovea reflection a measure of MPOD can be calculated.

4.6. Retinal Vessel Analyzer (RVA)

The dynamic vessel analyzer is a non-contact device used for measuring the retinal vessel diameter. In the present study, a fundus camera-based system with a single mode laser diode at a center wavelength of 670 nm will be used (Oculix Sarl, Arbaz, Switzerland) to tape a video of the retinal fundus. Then a computer-software detects the borders of the retinal vessels and measures continuously the diameter at the marked position.

Flicker stimulation

For flicker stimulation a custom built device is used, stimulating with light flashes at a frequency of 8 Hz. Flicker was generated by focusing the light of a 150 W halogen light source on a rotating sector disc producing a square wave light pattern with a modulation depth of 100%. Using an optical fiber, flicker stimuli were delivered to the eye through the illumination pathways of the fundus camera of the RVA.

4.7. Laser Doppler Velocity (LDV)

In the present study, a fundus camera-based system with a single mode laser diode at a center wavelength of 670 nm will be used (Oculix Sarl, Arbaz, Switzerland). The principle of blood flow velocity measurement by laser Doppler velocimetry is based on the optical

Doppler effect. Laser light, which is scattered by moving particles (e.g., erythrocytes), is shifted in frequency. This frequency shift is proportional to the blood flow velocity in the retinal vessel.17, 18 The maximum Doppler shift corresponds to the centerline erythrocyte frequency. LDV measurements were always done in the same location on a main inferior temporal retinal vein.

4.7.1. Retinal blood flow

Calculation of retinal blood flow will be done with the results of the measurements of blood flow velocity and retinal vessel diameter.

4.8. Color Doppler Imaging (CDI)

Measurement of posterior ciliary artery and ophthalmic artery blood velocity will be performed using a Vivid 7 Pro Color Ultrasound device (GE Vingmed Ultrasound, Horten, Norway). A combined transducer with 7.0 MHz for the B-mode (intensity: 40 mW/cm2) and 5.0 MHz for the pulsed Doppler (intensity: 25 mW/cm2) will be used. The probe will be placed on the closed upper eyelid following the application of contact jelly (methylcellulose 2%). To minimize the exertion of pressure on the globe, the examiner will support his hand on the subject's forehead. Peak systolic blood velocity (PSV) and end diastolic blood velocity (EDV) will be determined. In addition Resistive Index (RI) will be calculated as (PSV-EDV)/PSV and the mean flow velocity (MFV) will be measured as the time mean of the spectral outline.

4.9. Oxymap T1 Retinal Oxymeter

The Oxymap T1 is a non-invasive device for measuring the relative retinal vessel oxygen saturation and relative vessel diameter. The Oxymap takes pictures of the retina with a funuds camera and the Oxymap T1 software (Oxymap Analyzer) analyses those images and provides information about the oxygen saturation of the retinal vessels.

4.10 Adverse events

An adverse event is any event during a clinical study, including intercurrent illness or accident, which impairs the well-being of the subject; it may also take the form of an abnormal laboratory value. The term adverse event does not imply a causal relationship with the study treatment.

All subjects experiencing adverse events - whether considered associated with the use of the study medication or not - will be monitored until symptoms subside and any abnormal laboratory values have returned to baseline, or until there is a satisfactory explanation for the changes observed, or until death, in which case a full pathologist's report will be supplied, if possible. All findings must be reported on an "Adverse event" page in the case record form.

All adverse events, including intercurrent illnesses, will be reported and documented as described below.

Adverse events are divided into the categories "serious" and "nonserious". This determines the procedure which must be used to report/document the adverse event (see below).

4.10.1. Definition of serious, non-serious adverse events and unexpected serious adverse reaction

A serious adverse event is:

- any event that is fatal or life-threatening
- any event that is permanently disabling
- any event that requires hospitalization
- any event that involves cancer, or congenital anomaly

Adverse events which do not fall into these categories are defined as nonserious.

Suspected Unexpected Serious Adverse Reaction

A SUSAR is an undesired event, which is unexpected, serious and probably causal related to the test drug and therefore, be constituted as side effect.

Criteria:

- any SAE
- causal to study drug
- not yet listed in the investigator´s brochure

4.10.2. Reporting/Documentation of SAE, AE and SUSAR

Adverse events are collected by spontaneous reporting. Nonserious adverse events and SUSARs are documented on an "Adverse event" page in the case record form.

<u>Serious adverse events</u>

All serious adverse events which occur during this study, whether considered to be associated with the study medication or not, must be documented on an "Serious Adverse event" page in the case record form.

All serious adverse events will be reported to the Austrian health authorities and the ethics committee once per year using the “Development Safety Update Report”–form, as stipulated by Austrian Arzneimittelgesetz and EC-GCP guidelines.

<u>Nonserious adverse events</u>

These are to be documented on an "Adverse event" page in the case record form.

<u>SUSARs</u>

All serious adverse events will be reported immediately to the Austrian health authorities and the ethics committee as stipulated by Austrian Arzneimittelgesetz and EC-GCP guidelines.

4.10.3. Assessment of severity

Regardless of the classification of an adverse event as serious or nonserious (see above), its severity must be assessed as mild, moderate or severe, according to medical criteria alone:

mild = does not interfere with routine activities, acceptable
moderate = interferes with routine activities
severe = impossible to perform routine activities, considered as unacceptable by the physician, requires treatment, requires discontinuation of study, or has residual effect.

It should be noted that a severe adverse event need not be serious in nature and that a serious adverse event need not, by definition, be severe. Regardless of severity, all serious adverse events must be reported on as above.

4.10.4. Withdrawal rules and premature termination of the study

Patients will be excluded from the study:

in cases of serious adverse events
if the patient withdraws his/her consent to the study
in cases of endophthalmitis, retinal detachment, uveitis and cataract formation due to intravitreal injection
if the principal investigator deems the participation of a patient in this study not to be in his/her best interest

The principal investigator has the right to close this study at any time. The IEC and the competent regulatory authority must be informed within 15 days of early termination.
The trial or single dose steps will be terminated prematurely in the following cases:

- If adverse events occur which are so serious that the risk-benefit ratio is not acceptable.
- If the number of dropouts is so high that proper completion of the trial cannot realistically be expected

If a patient is excluded from the study all pieces of information until termination will be for statistical analysis, unless the patient withdraws consent for analysis.

The entire study should be discontinued under following condition:

If there are indications that the safety of study participants is no longer guaranteed
If the study question (s) is already clearly answered because of interim analysis (are)
If inadequate recruitment rate to graduate, which does not seem possible.

The study coordinator ensures that the sponsor will be informed immediately of the termination of the clinical trial by the examiner. The study coordinator will ensure that the relevant and affected parties ethics committees and the local authorities concerned receive an updated list of test centers.

4.11. Data handling procedures

An electronic case record form (eCRF) will be completed for each patient. The entries will be checked by trained personnel and any errors or inconsistencies will be checked by the electronic data capture (EDC) system. The patient-log of the pre-study screening examination will be documented in the study master file.

4.12. Biometric methods

4.12.1. Biometric methods – statistic planning and outcome variables

Statistical planning was done for alpha of 0.05 and a statistical power of 0.8 with an expected drop-out rate of 10% of the participating patients. Main parameter for sample size calculation was visual acuity, the end point being a loss of 15 letters in visual acuity. For a sample size of 50 patients the probability is 80 percent that the study will detect a treatment difference at a two-sided 0.05 significance level using an paired t-test, if the true difference between treatments is 1 times the standard deviation.

Anova and the paired t-test will be used to investigate the longitudinal changes in visual acuity, the central retinal thickness, retinal vessel diameters, retrobulbar flow velocities, retinal blood flow, macula pigment optical density. A p-value ≤ 0.05 is considered as statistical significant. For multiple t-testing a correction according to Bonferroni-Holmes will be performed.

Descriptive analysis will be performed for patient´s demographic data, furthermore, chi²-Test will be used for nominal parameters.

4.12.2. Biometric methods - Adverse events/Safety investigations

All adverse events will be properly listed and an appropriate method will be used to summarize the data.

5 ETHICAL AND LEGAL ASPECTS

The study will be carried out in accordance with the EU-GCP guideline and according to the Austrian Arzneimittelgesetz 1993. The study will be performed in accordance with the guidelines of the Declaration of Helsinki (1964), including current revisions.

5.1. Informed consent of subject

Before being admitted to this study, the subject must have consented to participate after the nature, scope and possible consequences of the clinical study have been explained in a form understandable to him (cf. Appendix).

The subject must give consent in writing. The subject's consent will be confirmed by the signatures of the investigator and a witness.

5.2. Acknowledgment/approval of the study and trial registration

Before the start of the study, the study protocol will be submitted to the Ethics Committee of Medical University of Vienna. Before initiation, the study will be registered at an open-access online database (www.clinicaltrials.gov). Furthermore the study will be registered at Austrian health authorities: BASG-Österreich as AMG-Study.

5.3. Insurance

An insurance covering all medical and diagnostic procedures of this study is provided for all patients at an insurance carrier.

5.4. Confidentiality

All subject names will be kept secret in the investigator's files. Subjects will be identified throughout documentation and evaluation by the number allotted to them during the study. The subjects will be told that all study findings will be stored and handled in strictest confidence.

5.5. Monitoring

Monitoring according to ICH GCP will be carried through by Univ. Prof. Dr. Michael Georgopoulos (Department of Ophthalmology, Medical University of Vienna, Austria). As required by GCP, at least 3 monitoring visits are scheduled - an initiation visit, one routine visit and a close out visit after the last patient has finished the study.

6 DOCUMENTATION AND USE OF STUDY FINDINGS

6.1. Documentation of study findings

All findings collected during the study will be entered on the case record forms. All entries in the case report forms will be made legibly in black ink. If corrections are made to entries in the case record form, the words or figures will be ringed and a single stroke drawn through them. The correct value will be entered beside the old entry and date and the correction will be initialed. Incorrect entries must not be covered with correcting fluid, or obliterated, or made illegible in any way. Case report forms will be completed immediately after the final examination.

The medical records upon which the case report form is based will be kept for at least 15 years.

6.2. Use if study findings

The findings of this study will be published by the investigators in a scientific journal and presented at scientific meetings. The manuscript will be circulated to all co-investigators before submission.

7 PROTOCOL AMENDMENTS

If any modifications (such as selection of additional variables) become necessary or desirable, these will be documented in writing; major changes require the approval of all investigators and the ethics committee.

8. REFERENCES

1. **Bopp, S.** Subretinal hemorrhage: Natural course and staging. *Ophthalmologe.* 2012, 109, pp. 635-643.

2. **Hunter, MA and et al.** Retinal angiomatous proliferation: clinical characteristics and treatment options. *Optometry.* 2004, 75, pp. 577-588.

3. **Ritter, M and et al.** Effect of intravitreal ranibizumab in avascular pigment epithelial detachment. *Eye.* 24, pp. 962-968.

4. **Ciardella, AP and et al.** Polypoidal choroidal vasculopathy. *Surv Ophthalmol.* 2004, Vol. 49, pp. 25-37.

5. **CATT Research Group**. Ranibizumab and Bevacizumab for Neovascular Age-Related Macular Degeneration. NEJM. 2011, 364, S.1897-1908.

6. **Heier J.S. et al.** Intravitreal Aflibercept (VEGF Trap-Eye) in Wet Age-related Macular Degeneration. Ophthalmology. 2012, 119, S.2537-2548.
